# The Book World of Medicine and Science

**Published:** 1904-06-25

**Authors:** 


### The Book World of Medicine and Science.

The Therapeutics of Mineral Springs and Climates. By I. Burney Yeo, M.D., F.R.C.P. (London: Cassell and Co. 12s. 6d. net.)

A book by an independent writer of authority dealing with watering places and health resorts and the indications necessary to enable the busy practitioner to decide quickly where to advise his patient to go has been a long-felt want. Many works have been published at intervals, but none of them, so far as we have seen, has attempted to deal in a comprehensive manner with all the places or cures from which patients may derive benefit. Dr. Burney Yeo's book appears to us to very successfully supply what is wanted. It is divided into two parts with complete indices. The first is devoted to the classification and composition of mineral springs and their internal and external therapeutic action. The author takes care to remind his readers that the remedial action of mineral waters is largely dependent on such associated conditions as changes in diet, climate, exercise, society, and so forth, and points out that many mineral springs whose curative effects are in themselves insignificant, have been raised into importance by the intelligent and skilful necessary methods attending their use. A full account of these accessories is given under the various headings throughout the work. The value and curative action of the *quantity* of water taken has an importance that is often overlooked. There is no doubt that most people use but little water as a beverage, and we have known some who boasted that they never knew the taste of water except for toilet purposes. To such persons the drinking of large quantities of this important solvent, apart from its mineral constituents, must have, as the author shows, most important physiological and therapeutical effects. Dr. Burney Yeo has investigated the alleged effect of "sipping" in quickening the heart beat by abolishing the inhibitory action of the vagus. After repeated tests he has established what common sense would seem to suggest that this method has wholly negative results except in highly nervous subjects prone to the influence of suggestion. The great value of the first part of this book is that it enables the reader to readily form general ideas on the various groups of waters and health resorts, while the places themselves, with full details, including analyses of the more important waters, will be found conveniently arranged in alphabetical order. We note that with regard to the places which the author has probably visited himself and had opportunities of personally studying his views on the various questions involved are stated with a definiteness that cannot fail to be helpful, while with regard to others, and especially those where the peculiarities of the methods employed are the chief features, or where general experience has not guaranteed the results claimed, he contents himself with saying "it is believed" that such and such effects follow and leaves the final decision apparently to faith. The second part dealing with health resorts in Europe, Africa, and America, and sea voyages necessarily partakes somewhat of the nature of a guide-book. Generally speaking, this part is quite up to the standard of the rest of the work. It was, of course, impossible for the author to check the statements of pre-existing guides on every point, but the information, especially regarding the places he has visited is concise and reliable. The part relating to Spain seems, however, to fall short of this standard, probably because the author is evidently under the impression that as few English people have hitherto visited Spain they are likely to continue to avoid it. They will certainly not be encouraged to a visit by the accounts given of Malaga and Gibraltar, which, after a somewhat prolonged acquaintance with both places, appear to us to be inaccurate and not up to date. Malaga has wonderfully improved as a winter station during the past six or seven years, both as regards comfort, amusements, and accessibility, and the same may be said of Gibraltar, which affords a suitable stopping place, at certain seasons, for those on their way to its more favoured neighbour. Apart from this trifling drawback, we have no hesitation in recommending Dr. Burney Yeo's handbook as one likely to prove of very great use and interest both to medical men and the travelling public.

Materia Medica, Pharmacology, and Therapeutics; Inorganic Substances. By Charles D. F. Phillips, M.D., LL.D.Aberd. and Edin., F.R.S., and F.R.C.S.Edin. Third Edition. (London: Longmans, Green and Co. 1904. Pp. xiii. and 921. Price 21s.)

The practical value of Dr. Phillips' work is attested by the appearance of a third edition. In form and arrangement the book adheres to the traditions of an earlier generation, but it is none the less serviceable on that account. The prevailing note is perhaps that of a cheerful confidence in the efficacy of medicinal treatment, and though it may be difficult at times to share this confidence, it is noteworthy that a physician of long experience is not afraid to confess strong convictions regarding the value of numerous drugs. The practitioner who consults this book will certainly find many valuable suggestions in the department of therapeutics, and access to these is facilitated by a well-arranged index of diseases with references to the appropriate drug agents that may be employed in their treatment. There are very numerous quotations from the writings of various authorities, and the collection of these shows much industry, but some of them, in the light of more recent knowledge, are of little value. A useful feature of the book is an account of the leading spas and health resorts both at home and abroad. As a manual of practical therapeutics Dr. Phillips' work is entitled to the success which it has achieved.

Orthmann's Handbook of Gynæcological Pathology. Translated by C. Hubert Roberts, M.D., F.R.C.S., M.R.C.P. (London: John Bale, Sons and Daniellsson, Limited. 1904. Price 5s. net.)

All students of gynæcological pathology will be grateful to Dr. Roberts for translating Orthmann's well-known handbook. A work of this nature has long been required, and we accord a hearty welcome to the volume before us. The first section, consisting of 20 pages, deals with the hardening, cutting, and staining of tissues, and gives clear directions for the practice of all methods which the student is likely to require. The second and much larger section of the book is devoted to morbid histology, and gives a short, yet clear, account of the appearances met with in the various forms of disease to which the female reproductive organs are liable. The illustrations, 73 in number, are admirable both in their accuracy and clearness, and are worthy to rank with the beautiful drawings of museum specimens which are found in Dr. Roberts' own book; they are faithful reproductions of the appearances which the observer may see in a good section and are therefore of real value. Dr. Roberts has performed his share of the work with skill and judgment, he has written in English, and not, like so many translators, in Anglicised German. He has kept very close to the original, but one almost regrets that he has not added a few marginal notes; for instance:—"The adenomatous cysts of the ovary are epithelial new growths which arise from its superficial epithelium (Muller's epithelium) not apparently from the normal germ epithelium," is a statement which certainly requires further explanation to the English student. The paper is good and the printing is clear; we congratulate Dr. Roberts on the production of so excellent a manual and wish it the success it undoubtedly deserves.

